# Comparison of Platforms for Testing Antibody Responses against the *Chlamydia trachomatis* Antigen Pgp3

**DOI:** 10.4269/ajtmh.17-0292

**Published:** 2017-09-25

**Authors:** Sarah Gwyn, Gretchen Cooley, Brook Goodhew, Stephan Kohlhoff, Natalie Banniettis, Ryan Wiegand, Diana L. Martin

**Affiliations:** 1 IHRC, Inc. Contractor at the Centers for Disease Control and Prevention, Atlanta, Georgia;; 2 Division of Parasitic Diseases and Malaria, Centers for Disease Control and Prevention, Atlanta, Georgia;; 3 State University of New York Downstate Medical Center, Brooklyn, New York

## Abstract

Antibody responses to *Chlamydia trachomatis* (CT) antigens may be useful tools for surveillance of trachoma by estimating cumulative prevalence of infection within a population. Data were compared from three different platforms—multiplex bead array (MBA), enzyme-linked immunosorbent assay (ELISA), and lateral flow assay (LFA)—measuring antibody responses against the CT antigen protein plasmid gene product 3 (Pgp3). Sensitivity was defined as the proportion of specimens testing antibody positive from a set of dried blood spots from Tanzanian 1–9-year olds who were positive for CT nucleic acid of all nucleic acid amplification test (NAAT)-positive individuals (*N* = 103). The sensitivity of the LFA could not be determined because of the use of dried blood spots for this test; this specimen type has yet to be adapted to LFA. Specificity was defined as the proportion of sera from U.S. and Bolivian 1–9-year olds that had previously tested negative by the *Chlamydia* microimmunofluorescence (MIF) assay testing negative to Pgp3-specific antibodies (*N* = 154). The sensitivity for MBA and ELISA was the same—93.2 (95% confidence interval [CI]: 88.3–98.1). Specificity ranged across platforms from 96.1 (95% CI: 91.8–98.2) to 99.4% (95% CI: 98.2–100). ELISA performance was similar regardless of whether the plates were precoated or freshly coated with antigen. Sensitivity and specificity of control panels were similar if the cutoff was determined using receiver operator curves or a finite mixture model, but the cutoffs themselves differed by approximately 0.5 OD using the different methodologies. These platforms show good sensitivity and specificity and show good agreement between tests at a population level, but indicate variability for ELISA outcomes depending on the cutoff determination methodology.

## INTRODUCTION

The bacterium *Chlamydia trachomatis* (CT) is the causative agent of the eye disease trachoma. Diagnosis of trachoma involves examining the upper tarsal conjunctiva for signs of trachomatous inflammation—follicular, requiring that at least five follicles of 0.5 mm be present in at least one eyelid to meet WHO criteria.^1^ Trachoma is estimated to account for 1.9 million cases of visual impairment globally.^2^ Through an integrated approach to disease control including Surgery, mass Antibiotic distribution, and Facial cleanliness and Environmental improvement (the SAFE strategy), the WHO alliance for the Global Elimination of Trachoma by 2020 aims to eliminate trachoma as a public health problem.^3^ Antibody responses to CT antigens have limited diagnostic value because of the longevity of the antibody response compared with the relative time of infection with CT.^4–7^ However, the seroprevalence of anti-CT antibodies may be useful in determining the cumulative risk of infection within young children in a population. Serological surveys have the potential to be useful for surveillance of trachoma in districts that have achieved the elimination targets.

The CT protein plasmid gene product 3 (Pgp3) is an immunodominant antigen in urogenital CT infection^8^ and has been used in serosurveillance studies for urogenital *Chlamydia*^9^ and trachoma.^6,10,11^ We have developed assays to detect Ab to Pgp3 on three separate platforms: multiplex bead array (MBA), enzyme-linked immunosorbent assay (ELISA), and lateral flow assay (LFA).^12^ Each platform has advantages in different usage scenarios. MBA allows for integration of serologic surveillance of a number of diseases in a single well using less than a drop of blood, ELISA requires less technical capacity than MBA but provides semiquantitative data, and the LFA is field-deployable and lower cost for low-resource settings but provides only dichotomous data. Having tests for Pgp3-specific antibodies available on multiple platforms offers a choice to country surveillance programs for testing based on country capacity and surveillance needs. However, multiplatform availability is only useful if the data obtained are comparable using any of the three different platforms.

In the current study, we report the sensitivity of MBA and ELISA using a set of specimens from individuals with ocular swab specimens positive by nucleic acid amplification tests (NAATs) and specificity of the MBA, ELISA, and LFA against a panel of pediatric specimens from nonendemic countries and evaluate the characteristics of the ELISA assay using different cutoffs and plate preparation approaches.

## METHODS

### Specimens.

Positive reference samples came from children in a trachoma-endemic district of Tanzania that had tested positive for ocular infection (*N* = 103, Wilson et al., unpublished data,^5,11^). Negative reference samples came from 4–9-year olds from three communities in central Bolivia collected as part of a study of Chagas disease (*N* = 81) and from a group 1–9-year olds (*N* = 74) from Brooklyn, NY.^13^ For comparison of precoated and freshly coated ELISA plates, serum samples were collected from all ages in a community in Nepal as part of trachoma serosurveillance study (*N* = 424). These were previously tested by MBA for antibodies against the CT antigen Pgp3 (S. Gwyn et al., unpublished data). Specimens included in the comparison of precoated and freshly coated ELISA plates (*N* = 748) were intentionally selected to represent a broad distribution of intensity of antibody responses and were comprised of all specimens previously mentioned as well as a specimen set collected from trachoma-endemic communities in Nepal (S. Gwyn et al., unpublished data). Table 1 indicates the various sample sets and sample set combinations used for each analysis and comparison.

**Table 1:** Sample sets used for the analyses and comparisons shown in this study

Sample set	Analysis				
	Sensitivity testing	Specificity testing	Platform comparison	Precoated vs. freshly coated plates—ELISA	ROC analysis
Nepal samples of all ages	0	0	424	424	0
Tanzanian samples of all ages for ROC—previously classified as Pgp3 antibody-positive or negative by MBA	0	0	0	66	66
Tanzanian samples from PCR-positive children	103	0	0	103	0
MIF (+) nonendemic children	0	0	1	1	0
MIF (−) nonendemic children	0	154	154	154	0
Total	103	154	579	748	66

ELISA = enzyme-linked immunofluorescence assay; MBA = multiplex bead array; MIF = microimmunofluorescence assay; PCR = polymerase chain reaction; Pgp3 = protein plasmid gene product 3; ROC = receiver operator characteristics. Each sample set is also described in the Methods Section. Note that there were some analyses that used a combination of sample sets.

### Ethics.

Samples were collected under approved protocols. For the Tanzanian sample set, approval was obtained from the Tanzania National Institute of Medical Research (NIMR), with U.S. Centers for Diseases Control and Prevention (CDC) Human Subjects Office Institutional Review Board relying on the NIMR approval. For Bolivian samples, approval was obtained from the Asociacion Beneficia PRISMA and from CDC. The samples were stripped of identifying information after these studies were terminated, and additional testing conducted in accordance with consents obtained for each study. Samples from Brooklyn were obtained anonymously; approval was obtained from the local institutional review boards (protocol #412878) and CDC determined that the use of these samples was not considered as human subjects research.

### Antigen preparation.

The selection and isolation of the CT antigen Pgp3 has been previously described.^6^ Briefly, XL1 Blue bacterial cultures containing Pgp3 (Pgp3 cloned from serovar E) in a pGEX6 (Amersham Biosciences, Piscataway, NJ) vector system were grown to an optical density of 0.7–0.8, induced with 0.2 MM IPTG, harvested after 2 hours, and the Pgp3-GST fusion protein purified over a glutathione Sepharose 4B affinity column (GE Healthcare, Piscataway, NJ).

### Multiplex bead array.

Six millimeter filter paper extensions (CellLabs, Sydney, Australia) calibrated to hold 10 μL of dried blood were placed in 2,000 μL of PBS containing 0.5% BSA, 0.05% Tween 20, 0.02% sodium azide, 0.5% polyvinyl alcohol, 0.8% polyvinylpyrrolidone, and 0.5 w/v *Escherichia coli* extract (to block binding of anti- *E. coli* antibody to beads and lower background levels) designated as PBN1 to elute serum. Serum was diluted 1:400 in PBN1 and incubated with microbeads coupled to Pgp3. Excess serum was washed off and bound antibody detected with an antihuman IgG (Southern Biotech, Birmingham, AL) and antihuman IgG4 (Southern Biotech) biotinylated detection antibody, and bound secondary antibody was detected using streptavidin conjugated to phycoerythrin (PE) (Invitrogen, Carlsbad, CA). Plates were read on a BioPlex 200 instrument (BioRad, Hercules, CA). The fluorescent signal emitted by bound PE was converted to a median fluorescence intensity (MFI) with background from the blank (PBN1 alone) subtracted out (MFI-BG).

### Enzyme-linked immunoassay.

Immulon 2HB plates (Thermo Fisher Scientific, Waltham, MA) were coated with 500 ng/mL of recombinant Pgp3 antigen (50 μL/well) in NaCO_3_ (pH 9.6) overnight at 4°C. Control and experimental sera were diluted 1:50 in PBS containing 0.3% Tween 20 (PBST) and 5% milk (PBST milk) the day before the assay and stored overnight at 4°C. On the day of the assay, unbound antigen was washed from the plates with 200 μL of PBST, and then, the plates were blocked with PBST for 1 hour at 4–10°C. Blocking solution was discarded, then diluted samples (50 μL) were added to the plates and the plates were incubated at room temperature on an orbital shaker for 2 hours. The samples were decanted and the plates washed four times with PBST. Antihuman IgG (Southern Biotech) diluted 1:10,000 in PBST (50 μL) was added to each well and incubated at room temperature on an orbital shaker for 1 hour. Plates were washed four times with PBST, then 50 μL of tetramethylbenzidine (TMB) developing reagent (3, 3′, 5, 5′-Tetramethylbenzidine, KPL, Gaithersburg, MD) was added to each well. After the experimentally determined time (see below), 50 μL 1N H_2_SO_4_ was added per well to stop the reaction. The plates were read immediately at 450 nm on a microplate reader (BioTek Synergy HTX, BioTek, Winooski, VT). To prepare precoated plates, the plates were coated with antigen as described above. The next day, the plates were washed four times with PBST, and then 100 µL of StabilCoat^®^ (SurModics, Eden Prairie, MN) was added to each well, shaken for 30 minutes, excess buffer removed, and the plates were dried in a vacuum oven at 30–40°C for 4 hours. Dried plates were stored in foil packages with a 1 g silica desiccant pack at 4°C. Before running the ELISA, the dried plates were removed from storage, the samples were added to plate, and the procedure was carried out as described above.

For plate quality control and sample normalization, we established a set of quality control measures within the protocol. We created a set of internal controls derived from serum testing positive for anti-Pgp3 antibodies which were diluted to create a set of standards denoted 1,000 units (U), 200 U, 100 U, and 50 U. Each standard is included on each plate, along with negative control serum, and PBST-milk for blank. Optical density for each sample with the blank value subtracted is normalized against the 200 U standard. Before running study samples, each laboratory should determine the optimal development time for the TMB developing reagent. This is done by running a plate with duplicate rows of the 1,000 U, 200 U, normal human serum (NHS), and blank wells, staggering TMB addition in each column from 4 to 15 minutes in 1 minute increments (4 minutes developing time in Column A, 5 minutes in Column B, 6 minutes in Column C, etc.). The optimal TMB incubation time is then chosen based on the following criteria: 1) the 200 normalization standard should be within 20% of an OD of 1.0 (i.e., 0.8–1.2 OD is acceptable), 2) the range between the 1,000 control and NHS should be > 1.5 OD, and 3) the ratio between the 1,000 control and NHS should be > 10. If several consecutive time points meet these criteria, a time point in the middle of this range is chosen. After TMB optimization, a series of 10 plates are run on at least five different days to set baseline acceptable parameters for controls. The ELISA protocol has been used in previous publications.^14,15^

### Lateral flow assay.

Pgp3 LFAs were constructed as previously described.^12^ Serum (10 µL) was applied to the sample applicator port, followed by 200 µL of chase buffer (10 mM PBS, 0.3% Tween 20) added to the buffer applicator port, forcing sample migration onto the conjugate pad. Any antibodies specific for Pgp3 in the sample bind to the Pgp3-gold conjugate and continue to travel up the nitrocellulose membrane. Anti-Pgp3 antibodies bound to the Pgp3-gold conjugate then bind to Pgp3 on the test line, producing a visible signal by accumulation of gold particles on the test line. SA-gold conjugate (Arista Biologicals, Allentown, PA) bound to BSA-biotin (Arista Biologicals) on the control line produces a signal irrespective of the presence of anti-Pgp3 antibodies in the sample. Excess liquid is absorbed by the absorbent pad. LFAs were read after 30 minutes.

### Microimmunofluorescence assay.

Pediatric specimens used for specificity testing were confirmed to be negative for antibodies against chlamydial antigens using microimmunofluorescence (MIF) assay (Focus Diagnostics, Cypress, California) according to manufacturer’s instructions. Briefly, the samples were diluted 1:16 in 1× PBS and added to each well on the slide. The slides were incubated for 30 minutes at 37°C and then rinsed with 1× PBS. After the slides were dry, 25 µL of anti-IgG conjugate was added to each slide well, incubated for 30 minutes at 37°C and then washed and dried. Mounting medium was applied on the slide and covered with a coverslip. The slides were viewed at a magnification of ×400 on a fluorescence microscope for classification as anti-CT antibody positive or negative. Positive controls were included in each run.

### Cutoff determination for MBA and ELISA.

For the MBA, the cutoff for seropositivity was determined using receiver operator characteristic (ROC) curve analysis. A panel of 66 samples previously classified as positive or negative for Pgp3 antibodies by MBA were run as positive and negative control panels. An MFI-BG value of 804 was established as the low-limit value for positivity.

For the ELISA, four different cutoffs were determined. The ROC curve analysis generated ODNorm cutoffs of 0.983 for freshly coated plates and 1.104 for pre-coated plates A fixed finite mixture model^14,15^ to determine the intersection of two distributions within the sample data of the 748 samples used for ELISA comparisons generated ODNorm cutoffs of 0.574 for freshly coated plates and 0.660 for precoated plates.

### Statistical analyses.

Sensitivity was defined as the proportion of NAAT-positive control specimens testing positive for antibody against Pgp3 from a set of dried blood spot specimens from 103 Tanzanian 1–9-year olds who had had ocular swabs that tested positive for CT nucleic acid by NAAT^6,11^ (Wilson et al., unpublished data). Sensitivity testing was not conducted on the LFA because the samples were dried blood spots, and these provide high background on the LFA which prohibits distinction of a positive test line. Specificity was defined as the proportion of serum specimens testing negative for antibody against Pgp3 from a set of serum specimens from U.S. and Bolivian 1–9-year olds testing negative by the *Chlamydia* MIF assay (*N* = 154), and 95% confidence intervals (CIs) were reported.

Normalized ODs from precoated and freshly coated plates were compared using linear regression analysis and Bland–Altman plots,^16^ which calculate the differences between ODs for paired specimens and the average of the two measures. Analyses were done using GraphPad Prism (v. 6.00, GraphPad Software, La Jolla, CA).

Differences in normalized OD of precoated plates after a change in coating buffer was determined using analysis of variance.

## RESULTS

**Figure 1.**
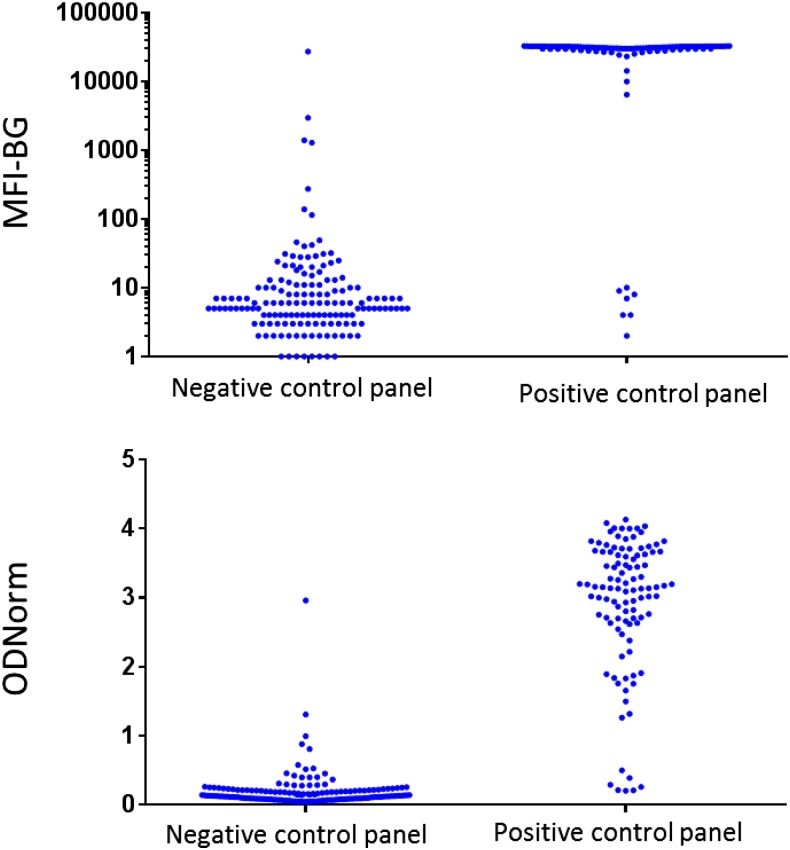
Intensity of anti-Pgp3 antibody responses of negative and positive control panels. Data shown represent antibody responses against Pgp3 measure on MBA (top panel) or ELISA (bottom panel). Each dot represents an individual specimen. Top panel shows MFI-BG on the *y* axis, bottom panel shows ODNorm on *y* axis. Negative control panel are MIF-negative sera from children in nonendemic countries (*N* = 154 as outlined in Table 1) and positive control panel are sera from children in trachoma-endemic regions of Tanzania with a CT-positive ocular swab (*N* = 103 as outlined in Table 1).

Sensitivity was 93.2% (95% CI: 88.3–98.1) for the MBA (Table 2). Specificity was 97.4% (95% CI: 94.9–99.9) for the MBA and 99.4% (95% CI: 98.2–100) for the LFA. When using the cutoff determined from ROC analysis for the ELISA, the sensitivity of the assay when using either precoated or freshly coated plates was 93.2% (95% CI: 88.3–98.1). The specificity was 97.4% (95% CI: 94.9–100%) for precoated plates and 98.1% (95.9–100) for freshly coated plates. When using the mixture model to determine the ELISA cutoffs, the sensitivity for freshly coated and precoated plates was 93.2% (95% CI: 88.3–98.1%). The specificity for precoated plates was 96.8% (95% CI: 92.6–98.6%) and the specificity using freshly coated plates was 96.1% (95% CI: 91.8–98.2%). The MFI-BG and ODNorm for the positive and negative control panels used in sensitivity and specificity analysis, respectively, are shown in Figure 1.

**Table 2:** Specificity of MBA, ELISA, and LFA tests measuring antibodies to the CT antigen Pgp3

	Cutoff determination	Specificity (95% CI)	Sensitivity (95% CI)
MBA	ROC	97.4% (94.9–100)	93.2% (88.3–98.1)
ELISA (freshly coated)	ROC	98.1% (95.9–100)	93.2% (88.3–98.1)
ELISA (precoated)	ROC	97.4% (94.9–100)	93.2% (88.3–98.1)
ELISA (freshly coated)	MM	96.1% (91.8–98.2)	93.2% (88.3–98.1)
ELISA (precoated)	MM	96.8% (92.6–98.6)	93.2% (88.3–98.1)
LFA	Visual inspection of test line	99.4% (98.2–100)	n.d.

CI = confidence interval; CT = *Chlamydia trachomatis*; ELISA = enzyme-linked immunofluorescence assay; LFA = lateral flow assay; MBA = multiplex bead array; MIF = microimmunofluorescence assay; MM = mixture model; NAAT = nucleic acid amplification test; n.d. = not done; Pgp3 = protein plasmid gene product 3; ROC = receiver operator characteristic. Specificity is shown as percentage of pediatric MIF-negative samples testing negative by each assay, with 95% CIs in parentheses. Sensitivity is shown as percentage of specimens from NAAT-positive individuals testing positive by each assay, with 95% CIs in parentheses.

The overall agreement between the positive tests using the three assays was highest when using the mixture model to determine the ELISA cutoff, as 96.2% (95% CI: 92.7–98.1) of samples positive by MBA were also positive by ELISA and LFA (Table 3). This was the same regardless of if the ELISA plates were freshly coated or precoated with antigen. When ROC was used for ELISA cutoffs, the agreement dropped to 82.7% (95% CI: 77.1–87.1) for precoated plates and 86.1 (95% CI: 80.9–90.1) for freshly coated plates (Table 3). The agreement between negative tests was approximately 90% for the three assays, ranging from 87.3% (95% CI: 83.2–90.6) when using freshly coated plates and the mixture model for cutoffs to 93.1% (95% CI: 89.6–95.5) when using the precoated ELISA and ROC analysis for cutoffs (Table 3).

**Table 3:** Agreement between three immunoassays measuring antibody responses against the CT antigen Pgp3

		MBA-positive (*N* = 237)		MBA-negative (*N* = 332)	
		LFA positive (% [95% CI])	LFA negative (% [95% CI])	LFA positive (% [95% CI])	LFA negative (% [95% CI])
ELISA-fresh-ROC	Positive	86.1 (80.9–90.1)	0.8 (0.14–3.3)	1.8 (0.7–4.1)	2.1 (0.9–4.5)
	Negative	10.5 (7.1–15.3)	2.5 (1.0–5.7)	3.3 (1.7–6.0)	92.8 (89.3–95.2)
ELISA-fresh-MM	Positive	96.2 (92.7–98.1)	1.7 (0.5–4.6)	3.6 (2.0–6.4)	7.5 (5.0–11.0)
	Negative	0.4 (0.02–2.7)	1.7 (0.5–4.6)	1.5 (0.6–3.7)	87.3 (83.2–90.6)
ELISA-precoated-ROC	Positive	82.7 (77.1–87.1)	0.8 (0.14–3.3)	1.2 (0.4–3.3)	1.8 (0.7–4.1)
	Negative	13.9 (9.9–19.1)	2.5 (1.0–5.7)	3.9 (2.2–6.8)	93.1 (89.6–95.5)
ELISA-precoated-MM	Positive	96.2 (92.7–98.1)	1.3 (0.3–4.0)	3.3 (1.7–6.0)	3.9 (2.2–6.8)
	Negative	0.4 (0.02–2.7)	2.1 (0.8–5.1)	1.8 (0.7–4.1)	91.0 (87.2–93.7)

CI = confidence interval; CT = *Chlamydia trachomatis*; ELISA = enzyme-linked immunosorbent assay; LFA = lateral flow assay; MBA = multiplex bead array; MM = fixed mixture model; Pgp3 = protein plasmid gene product 3; ROC = receiver operator characteristic. A set of 579 samples (provenance of samples detailed in Table 1) were tested for antibody responses using MBA, ELISA, and LFA. Ten samples were inconclusive on LFA and those data were excluded from analysis (final *N* = 569). Data shown are percentage of samples that fall into the indicated classification with 95% CIs shown in parentheses.

**Figure 2.**
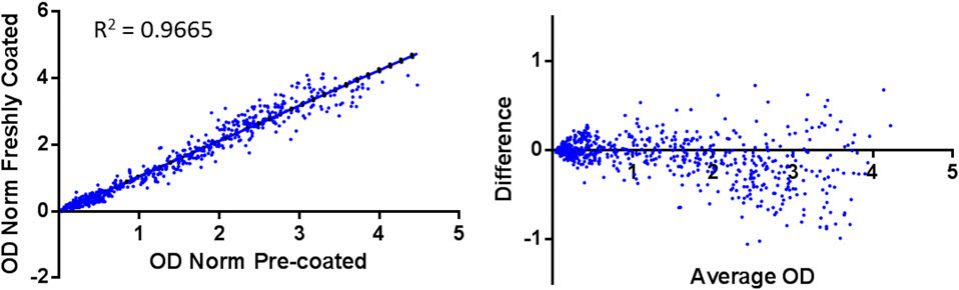
Comparison of ELISA data from plates freshly coated or precoated with Pgp3 antigen. Serum samples (*N* = 748) were run on Immulon H2B plates coated with Pgp3 antigen 16 hours previous (overnight, “ODNorm freshly coated”) or previously coated with antigen and dried in StabilCoat^®^ buffer (ODNorm precoated). Left plot shows linear regression analysis of the normalized OD (ODNorm) of the same specimens run on precoated plates (*x* axis) and freshly coated plates (*y* axis). The R square value indicating goodness of fit is shown. The right panel shows a Bland–Altman plot calculating difference (ODNorm of precoated plate—ODNorm of freshly coated plate; *y* axis) vs. the average of the normalized OD (*x* axis). *y* axis limits are −1.5 to 1.5.

Out of 748 samples analyzed by ELISA, 728 (97.3%, 95% CI: 95.91–98.27) were classified the same (i.e., positive or negative) regardless of the plate sensitization method when using the cutoffs determined by ROC analysis. When using the mixture model to determine ELISA cutoffs, 732 out of 748 samples (97.9%, 95% CI: 96.55–98.68) were classified the same. The absorbance readouts were similar based on regression analysis (goodness of fit = 0.9665, Figure 2, left panel) and Bland–Altman plots (bias = −0.06451, Figure 2, right panel). When comparing agreement by methodology for cutoff determination, the agreement was 93.0% (696/748) for the precoated plates and 92.5% (692/748) for the freshly coated plates. Fifty-two samples (6.95%, 95% CI: 5.34–9.0) tested positive using the mixture model cutoff and negative using ROC cutoffs on precoated plates, and 56 (7.49%, 95% CI: 5.81–9.6) specimens tested positive using the mixture model cutoff and negative using ROC cutoffs on freshly coated plates (Table 4).

**Table 4:** Pgp3 ELISA classification of samples using two different cutoff methodologies

Pre-coated ELISA plates		
	Pos _MM_	Neg _MM_
Pos _ROC_	331	0
Neg _ROC_	52	365

Freshly coated ELISA plates		
	Pos _MM_	Neg _MM_
Pos _ROC_	343	0
Neg _ROC_	56	349

ELISA = enzyme-linked immunosorbent assay; Pgp3 = protein plasmid gene product 3; MM = cutoff estimated using mixture model; Neg = negative classification (equal to or below the estimated cutoff threshold); Pos = positive classification (above the estimated cutoff threshold); ROC = cutoff estimated using receiver operator characteristic curve. Numbers in each box represent N per classification out of 748 samples total tested by ELISA. Top panel shows data from ELISAs using precoated plates, bottom panel shows data from ELISAs using same samples on freshly coated plates.

**Figure 3.**
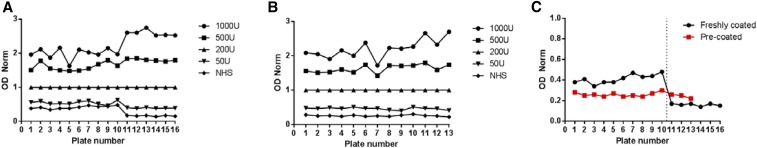
Control samples from all Pgp3 ELISA study plates. Normalized OD shown on *y* axis and plate number is on *x* axis. (**A**) Freshly coated plates. (**B**) Precoated plates. For (**A**) and (**B**), all control samples (top to bottom: 1000U, 50U, 200U, 50U, and NHS samples) are included. (**C**) NHS from freshly coated (circles) or precoated (red squares) plates. Vertical line indicates both a change in coating buffer for freshly coated plates and use of a new lot of precoated plates. Freshly coated plates 5, 7, and 10 failed QC and were rerun as plates 14, 15, and 16, respectively.

The full ELISA protocol dictates that ODs (raw and normalized) from controls be recorded in a single graph for observation of trends over time. These data for normalized ODs are shown in Figure 3A for freshly coated plates and Figure 3B for precoated plates. Figure 3C shows an overlay of the NHS curves from precoated and freshly coated plates, and the vertical line indicates a change in the batch of coating buffer used for freshly coated plates. A statistically significant drop in OD (*P* < 0.0001) was seen in the ODNorm of the NHS samples in freshly coated plates but not in precoated plates.

## DISCUSSION

Serosurveillance provides an opportunity for evaluating population-level exposure to the bacterium *C. trachomatis*. When integrated with age and disease epidemiology, Pgp3 antibody prevalence can give information about the burden of urogenital *Chlamydia* and trachoma in a population. We show here that three different platforms to assess antibody responses to Pgp3—MBA, ELISA, and LFA—show high specificity, that the MBA and ELISA show good sensitivity, and that there is good agreement between the three tests at a population level, with a few notable exceptions.

The generally high level of agreement across results obtained from the three platforms may seem unsurprising because of the use of the same target (recombinant Pgp3), but the nature of the readouts (i.e., a visible test line for the LFA compared with signal amplification in the ELISA and MBA assays) and the different volumes of sample used in each test had the potential to affect the relative sensitivities of the assays. The tests required different volumes of serum as follows: 10 µL neat serum for LFA, 50 µL of a 1:50 dilution of serum in ELISA, and 50 µL of a 1:400 dilution of serum in the MBA. In addition, *E. coli* extract is used only in MBA, not the ELISA, and this may have improved the specificity of this test by decreasing the nonspecific reactivity associated with bacterial proteins potentially coeluted with the recombinant Pgp3.

The method for determining the cutoff has the potential to have a significant impact on the estimated prevalence of antibody positivity within a population.^15^ Here, we compared the finite mixture model to ROC analysis, in which the cutoff was optimized for both sensitivity and specificity against the ROC panel. The ELISA cutoff using ROC was higher for both precoated and freshly coated plates compared with the cutoff obtained using the mixture model, which resulted in a slightly lower specificity. The differences in cutoffs between the two methodologies were more pronounced than what previous studies have shown.^15^ The ROC curve allows tuning in favor of maximizing specificity and/or sensitivity, whereas using the third standard deviation in the mixture model estimates specificity within the 97.5% CI. An advantage of using the mixture model is that there is the potential for a higher background level in “negative” populations from different countries or regions^6^ based on the varying range of exposure to infectious agents the immune system is subjected to in different geographic settings. The mixture method also relieves the requirement to have previously categorized sample sets available and saves time and money by not having to run additional ROC plates ahead of the study. Should Pgp3 antibody-based testing prove a viable option for trachoma surveillance, development of Pgp3-specific positive-control monoclonal antibody will be useful for quality control and validation between different laboratories using this assay, similar to what has recently been developed for Ov16-based ELISA testing for onchocerciasis.^17^

A major limitation to evaluating the use of antibody-based surveillance for trachoma is the lack of gold standard for CT antibody positivity to validate the approach—in other words, a measure of who has ever had exposure sufficient to generate Ab responses. We therefore used ocular swab NAAT-positivity as a gold standard, but recognize that there may be reasons beyond antibody assay sensitivity as to why a NAAT-positive individual would be antibody negative, such as detection of an initial ocular CT infection before the development of an antibody response. The use of sera from infection-positive children as the positive reference set may also bias the results, as these children tend to have very high antibody responses.^5,6^ It is possible that as the infection wanes, the antibody response does as well, although currently available data suggest a long-lived antibody response even after treatment.^5^ Whereas further testing in a variety of geographic settings will be needed to be done to confirm these sensitivity estimates, a recent study in Kiritimati Island, Kiribati, showed 90% of NAAT-positive 1–9-year olds testing positive for Pgp3 antibodies (Cama et al, submitted for publication). Because antibodies measure historical exposure, we could not obtain a set of “negative” samples from trachoma-endemic communities and therefore used samples from children from countries not endemic for trachoma. Another limitation of our study is that we only have DBS available from children with ocular infection and not sera, so we were unable to test the sensitivity of the LFA in this analysis because the LFA does not, in its current form, work with DBS. Upcoming field trials of the LFA will offer an opportunity to evaluate the sensitivity of the LFA. Whereas determination of the sensitivity and specificity against a gold standard for trachoma would be ideal, latent class analysis as a means to determine sensitivity and specificity in the absence of a gold standard will be informative.

Historically, serological assays for CT proteins, of which measurement of Pgp3-specific antibodies has been a particular focus, have lacked sensitivity and specificity. The specificity and sensitivity of the tests described herein have been calculated using a set of pediatric serum samples as negative controls and children with NAAT-positive ocular swabs from trachoma-endemic villages as positive controls, respectively. The initial selection of Pgp3 for our MBA was based off the recognition of this antigen by serum from 96 of 100 women with confirmed urogenital CT infection and reactivity to CT-infected cultures (i.e., high-titer serum).^18^ In other studies of urogenital CT infection, the sensitivities of Pgp3 ELISAs have ranged from 44.2% (44.2% for men and 73.8% for women)^19^ to 82.9% (54.4% for men, 82.9% for women)^9^ against specimens from individuals testing positive for urogenital CT. Other studies evaluating Pgp3-specific antibodies do not report assay sensitivity.^8,20^ Our Pgp3 MBA has previously shown sensitivity of approximately 95% (58/61, 95% CI: 89.6–100).^5,6^ The data here show a comparable sensitivity of the MBA and ELISA. We suspect the higher sensitivity of our ELISA compared with that seen in earlier studies is not because of a better-performing assay but because of the different sample sets under evaluation. Children in trachoma-endemic communities likely receive multiple ocular infections per year,^4^ with the potential to continually boost the antibody response and result in long-lived antibody-producing plasma cells. The MFI of specimens from children with ocular infection (i.e., NAAT-positive ocular swabs) on MBA are uniformly high (> 25,000 MFI^5,6^), so these high-titer responses are more readily detected in an immunoassay. Efforts should be undertaken to share sample sets among different laboratories to compare the performance of the various “in-house” Pgp3 ELISAs on diverse sample sets.

The ELISA described here is already being used for operational research studies for trachoma surveillance^14,15^ and is currently being run in laboratories in Malawi and Ghana on sample sets exceeding 5,000 per country. Streamlining the ELISA is one way to support testing of samples in laboratories in trachoma-endemic countries, which often cannot fully commit their time to operational research. Providing precoated plates would be one way to minimize the workload, and the data shown here show that precoated, dried plates perform similarly to those coated the day before sample analysis. The data also suggest that precoated plates may be superior in lot-to-lot consistency in the background of the negative control serum over time (Figure 3). The precoat step also blocks the plates, so the samples are added directly to the plate, thereby saving significant time in the sample analysis.

Our evaluation of the performance of three platforms for testing antibody responses to the CT antigen Pgp3 demonstrates that they have similar specificities and sensitivities, suggesting that data obtained from different platforms will yield similar community- or population-level prevalence. Studies to evaluate the robustness of these assays and further comparing of these platforms for analyzing antibody responses to Pgp3 in data obtained from different countries and in tests performed in different laboratories are currently underway, and will be critical for evaluating these tests for potential use in trachoma surveillance.

## References

[1] Thylefors B Dawson CR Jones BR West SK Taylor HR, 1987 A simple system for the assessment of trachoma and its complications. Bull World Health Organ 65: 477–483. 3500800 PMC2491032

[2] WHO, 2014 WHO alliance for the elimination of blinding trachoma by the year 2020. Wkly Epidemiol Rec 92: 421–428. 25275153

[3] WHO, 1997 *Report of the First Meeting of the WHO Alliance for the Global Elimination of Trachoma.* Geneva, Switzerland: World Health Organization.

[4] Bailey R Duong T Carpenter R Whittle H Mabey D, 1999 The duration of human ocular *Chlamydia trachomatis* infection is age dependent. Epidemiol Infect 123: 479–486. 10694161 10.1017/s0950268899003076 PMC2810784

[5] Goodhew EB 2014 Longitudinal analysis of antibody responses to trachoma antigens before and after mass drug administration. BMC Infect Dis 14: 216. 24755001 10.1186/1471-2334-14-216 PMC4016634

[6] Goodhew EB Priest JW Moss DM Zhong G Munoz B Mkocha H Martin DL West SK Gaydos C Lammie PJ, 2012 CT694 and pgp3 as serological tools for monitoring trachoma programs. PLoS Negl Trop Dis 6: e1873. 23133684 10.1371/journal.pntd.0001873 PMC3486877

[7] Horner PJ Wills GS Reynolds R Johnson AM Muir DA Winston A Broadbent AJ Parker D McClure MO, 2013 Effect of time since exposure to *Chlamydia trachomatis* on *Chlamydia* antibody detection in women: a cross-sectional study. Sex Transm Infect 89: 398–403. 23430706 10.1136/sextrans-2011-050386

[8] Comanducci M Manetti R Bini L Santucci A Pallini V Cevenini R Sueur JM Orfila J Ratti G, 1994 Humoral immune response to plasmid protein pgp3 in patients with *Chlamydia trachomatis* infection. Infect Immun 62: 5491–5497. 7960130 10.1128/iai.62.12.5491-5497.1994 PMC303293

[9] Horner P Soldan K Vieira SM Wills GS Woodhall SC Pebody R Nardone A Stanford E McClure MO, 2013 *C. trachomatis* Pgp3 antibody prevalence in young women in England, 1993–2010. PLoS One 8: e72001. 23991024 10.1371/journal.pone.0072001 PMC3749119

[10] Martin DL 2015 Serology for trachoma surveillance after cessation of mass drug administration. PLoS Negl Trop Dis 9: e0003555. 25714363 10.1371/journal.pntd.0003555 PMC4340913

[11] Martin DL 2015 Serological measures of trachoma transmission intensity. Sci Rep 5: 18532. 26687891 10.1038/srep18532 PMC4685243

[12] Gwyn S Mitchell A Dean D Mkocha H Handali S Martin DL, 2016 Lateral flow-based antibody testing for *Chlamydia trachomatis*. J Immunol Methods 435: 27–31. 27208400 10.1016/j.jim.2016.05.008

[13] Banniettis N Thumbu S Szigeti A Chotikanatis K Braunstein M Gadir GAE Hammerschlag M Kohlhoff S, 2015 Seroprevalence of *Chlamydia trachomatis* (CT) in inner city children and adolescents: implications for vaccine development. Open Forum Infect Dis 2: 1569. 10.1097/OLQ.0000000000000683 28876302

[14] Cocks N 2016 Community seroprevalence survey for yaws and trachoma in the western division of Fiji. Trans R Soc Trop Med Hyg 110: 582–587. 27852877 10.1093/trstmh/trw069 PMC5155547

[15] Migchelsen SJ 2017 Defining seropositivity thresholds for use in Trachoma Elimination Studies. PLoS Negl Trop Dis 11: e0005230. 28099433 10.1371/journal.pntd.0005230 PMC5242428

[16] Bland JM Altman DG, 2003 Applying the right statistics: analyses of measurement studies. Ultrasound Obstet Gynecol 22: 85–93. 12858311 10.1002/uog.122

[17] Golden A 2016 Analysis of age-dependent trends in Ov16 IgG4 seroprevalence to onchocerciasis. Parasit Vectors 9: 338. 27296630 10.1186/s13071-016-1623-1 PMC4907250

[18] Wang J Zhang Y Lu C Lei L Yu P Zhong G, 2010 A genome-wide profiling of the humoral immune response to *Chlamydia trachomatis* infection reveals vaccine candidate antigens expressed in humans. J Immunol 185: 1670–1680. 20581152 10.4049/jimmunol.1001240

[19] Wills GS Horner PJ Reynolds R Johnson AM Muir DA Brown DW Winston A Broadbent AJ Parker D McClure MO, 2009 Pgp3 antibody enzyme-linked immunosorbent assay, a sensitive and specific assay for seroepidemiological analysis of *Chlamydia trachomatis* infection. Clin Vaccine Immunol 16: 835–843. 19357314 10.1128/CVI.00021-09 PMC2691054

[20] Donati M Laroucau K Storni E Mazzeo C Magnino S Di Francesco A Baldelli R Ceglie L Renzi M Cevenini R, 2009 Serological response to pgp3 protein in animal and human chlamydial infections. Vet Microbiol 135: 181–185. 18945555 10.1016/j.vetmic.2008.09.037

